# Life Lessons in Learning: Continuity & Change in Gerontological Education

**DOI:** 10.1093/geroni/igaf122.1239

**Published:** 2025-12-31

**Authors:** Rona Karasik

**Affiliations:** Saint Cloud State University, Saint Cloud, Minnesota, United States

## Abstract

The title of Robert Atchley’s introductory textbook Aging: Continuity & Change (1983,1987) reflects two enduring themes in gerontological education. On the one hand, it is essential, albeit daunting, to keep abreast of the rapidly emerging advances in age-related science, technology, health care and public policy. Change is inevitable and on-going preparation a necessity for gerontological educators. Ultimately, no two offerings of a particular gerontology course are ever exactly the same, nor should they be. On the other hand, there are also inescapable constants to contend with such as ageism, inequity, a lagging workforce, and the never-ending battle to attract students to the field of aging. This presentation examines the paradox of continuity and change in gerontological education over the past thirty years and considers potential trajectories for the next thirty.

